# Plasma exosomal miR‐339‐3p promotes myocardial remodeling in chronic heart failure by regulating USP25‐mediated DDX58 deubiquitination

**DOI:** 10.1002/ccs3.70090

**Published:** 2026-06-26

**Authors:** Guoqiang Jing, Ting Xu, Yuhong Ma

**Affiliations:** ^1^ Department of Cardiology Affiliated Hospital of Inner Mongolia Medical University Hohhot China; ^2^ Inner Mongolia Medical University Hohhot China; ^3^ Department of General Practice Affiliated Hospital of Inner Mongolia Medical University Hohhot China

**Keywords:** exosome, HF, miR‐339‐3p, myocardial remodeling, USP25

## Abstract

To explore the mechanism by which plasma exosome miR‐339‐3p regulates myocardial remodeling in heart failure (HF). Plasma exosomes were isolated from 5 patients with HF and 5 controls, and cell uptake was determined by transmission electron microscopy, western blot and PKH26 labeling. Angiotensin II treated AC16 cells to induce cell hypertrophy. Cell counting kit‐8 assay and flow cytometry were used to detect cell viability and apoptosis to evaluate the effect of HF‐exo. High‐throughput sequencing was performed on exosomal microRNAs to identify key differential mirnas (miR‐339‐3p), and database screening was used to verify downstream target proteins ubiquitin‐specific protease 25 (USP25) by dual‐luciferase reporter gene assay and reverse transcription‐quantitative polymerase chain reaction/western blot. Searchtool for the retrieval of interacting genes, the Cancer Genome Atlas and co‐immunoprecipitation were used to screen USP25‐interacting proteins (DDX58). Analysis revealed that inhibition of miR‐339‐3p completely reversed cardiomyocyte injury induced by HF‐exo. miR‐339‐3p directly targets USP25, down‐regulates its expression, and impairs its K48‐related DDX58 deubiquitination. Overexpression of USP25 can reverse myocardial injury induced by mir‐339‐3p, and DDX58 silencing can eliminate the protective effect of USP25. Plasma exosome miR‐339‐3p promotes myocardial remodeling in HF through the miR‐339‐3p‐USP25‐DDX58 axis and is a potential target for the diagnosis and treatment of HF.

## Graphical abstract

In the patients of chronic heart failure (HF), miR‐339‐3p derived from plasma exosomes can target ubiquitin‐specific protease 25, inhibiting its K48‐linked deubiquitination modification of DDX58, leading to increased ubiquitination and degradation of DDX58. This further exacerbates cardiomyocyte apoptosis and hypertrophy, ultimately promoting HF myocardial remodeling.

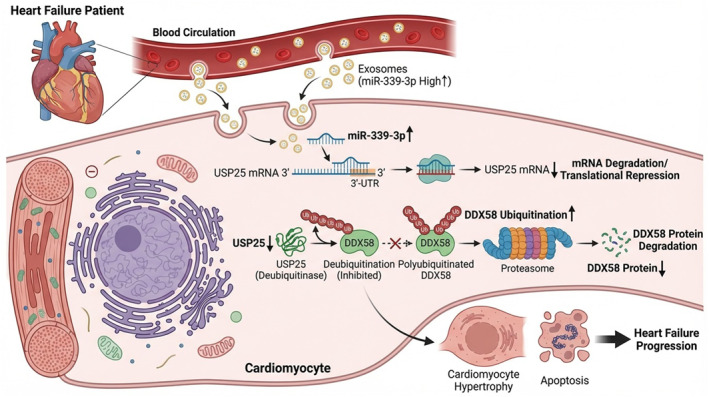

## 1 INTRODUCTION

Chronic heart failure (HF) represents the terminal stage in the progression of diverse cardiovascular diseases and constitutes the leading cause of mortality worldwide.^1^ Although recent advances in systemic therapies—including statins and antihypertensive agents—as well as cardiac‐targeted interventions have been developed and shown to significantly improve short‐term outcomes in cardiovascular disease, the incidence of HF remains high, and long‐term survival rates have not substantially improved.^2^, ^3^ Pathological myocardial remodeling has been identified as a critical pathophysiological substrate driving the progression of cardiovascular diseases toward HF. Central to this process are myocardial cell loss and impaired contractile function, accompanied by multiple pathological alterations, including inflammatory cell infiltration, myocardial fibrosis, mitochondrial dysfunction, and dysregulated intercellular signaling.^4^ Despite these insights, our understanding of the underlying molecular mechanisms remains incomplete; consequently, elucidating the key regulatory pathways governing pathological myocardial remodeling is an urgent priority to improve, reverse, or delay its progression and the subsequent development of HF.

Exosomes are extracellular vesicles with diameters ranging from approximately 30 to 150 nm, exhibiting a circular or cup‐shaped morphology enclosed by a lipid bilayer membrane.^5^ Functioning as critical mediators of intercellular communication, exosomes can selectively modulate cellular signaling by transporting bioactive molecules such as microRNAs and proteins. Previous studies have highlighted the involvement of exosomes in pathophysiological processes including myocardial ischemia, ischemia‐reperfusion injury, and angiogenesis. For instance, exosomal miR‐155‐5p has been reported to exacerbate myocardial ischemia–reperfusion injury by inhibiting NEDD4‐mediated CYP2D ubiquitination,^6^ whereas exosomal miR‐24, induced by remote ischemic preconditioning, exerts a protective effect against this injury.^7^ In patients with acute HF, exosomes have been demonstrated to promote endothelial angiogenesis through the miR‐126‐3p/TSC1/mTORC1/HEF‐1α signaling pathway.^8^ Nevertheless, whether plasma exosomal mRNAs in patients with HF participate in the regulation of myocardial remodeling, and the specific molecular mechanisms involved, remain unclear.

miR‐339‐3p, a microRNA previously implicated in cardiovascular diseases, has been closely associated with aortic aneurysmal dilatation in patients at high cardiovascular risk,^9^ and can promote vascular inflammation through upregulation of NFATc3 expression in vascular smooth muscle cells.^10^ Moreover, its expression level demonstrates a significant correlation with the N‐terminal pro‐B‐type natriuretic peptide (NT‐proBNP), a widely recognized biomarker of HF, suggesting that miR‐339‐3p may play a critical role in the pathophysiology of HF.^11^ However, the specific function and regulatory mechanisms of miR‐339‐3p in myocardial remodeling during HF remain largely unexplored.

Ubiquitination and deubiquitination modifications of proteins, mediated by the ubiquitin–proteasome system, constitute fundamental mechanisms regulating protein stability and function. Within this system, deubiquitinating enzymes (DUBs) exert precise control over the ubiquitination status of target proteins, thereby influencing cardiac pathophysiology. ubiquitin‐specific protease 25 (USP25), a member of the DUB family, has been demonstrated to play a pivotal regulatory role in cardiovascular and cerebrovascular diseases.^12^ For instance, in models of myocardial hypertrophy, USP25 is upregulated in both mouse cardiomyocytes and human hypertrophic heart tissue. It interacts with SERCA2a via its USP domain, removing K48‐linked ubiquitin chains to prevent protein degradation, thereby maintaining calcium homeostasis and mitigating cardiac hypertrophy.^13^ Additionally, USP25 confers neuroprotection in ischemic stroke by binding to TAB2 through its UIM2 domain, removing K63‐linked polyubiquitin chains, suppressing nuclear factor‐kappa B (NF‐κB)/mitogen‐activated protein kinase (MAPK) signaling, attenuating microglial neuroinflammation, and reducing ischemic damage. Notably, the observed upregulation of USP25 in ischemic penumbra microglia of stroke patients further highlights its potential as a therapeutic target.^13^

In the present study, bioinformatic analyses using TargetScan and miRDB databases suggested that USP25 may represent a downstream target of miR‐339‐3p. However, whether a direct regulatory relationship exists between miR‐339‐3p and USP25, and whether this interaction participates in myocardial remodeling in chronic HF, has not been experimentally confirmed. This research aimed to investigate the targeting relationship between plasma exosomal miR‐339‐3p and USP25, as well as the molecular mechanism by which USP25 modulates myocardial remodeling through deubiquitination of downstream target proteins. The results are expected to provide a theoretical foundation for the prevention and treatment of pathological myocardial remodeling in chronic HF.

## 2 MATERIAL AND METHODS

### 2.1 Cell culture and treatment

The human cardiomyocyte cell line AC16 (RRID: CVCL_4U18), derived from male human ventricular myocardial tissue, was obtained from the Cell Bank of the Chinese Academy of Sciences in March 2024.^14^ Cell line authentication was performed via short tandem repeat profiling by Genetic Testing Biotechnology Co., Ltd., confirming the absence of misidentification or cross‐contamination. Prior to all experiments, mycoplasma contamination was excluded using a mycoplasma detection kit (Beyotime).

AC16 cells were maintained in Dulbecco's modified eagle's medium (DMEM, iCell) supplemented with 10% fetal bovine serum and 1% penicillin/streptomycin at 37°C. To induce cardiac hypertrophy, cells were treated with 1 μmol/L angiotensin II (Ang II; Abmole) for 48 h. The hypertrophic model was validated by evaluating the protein expression of fetal cardiac markers, including A‐type natriuretic peptide (ANP), B‐type natriuretic peptide (BNP), and Myh7.

For experimental interventions, AC16 cells were assigned to the following groups: (1) Ang II group, treated with Ang II alone to serve as a baseline control for hypertrophy induction; (2) Ang II + HF‐exo + negative control (NC) inhibitor group, treated with Ang II in combination with plasma‐derived exosomes from HF patients (HF‐exo) and a NC inhibitor; and (3) Ang II + HF‐exo + miR‐339‐3p inhibitor group, treated with Ang II, HF‐exo, and a miR‐339‐3p‐specific inhibitor.

### 2.2 Patients and control recruitment

Five patients diagnosed with HF were enrolled from the Affiliated Hospital of Inner Mongolia Medical University. Diagnosis adhered to established clinical criteria, including symptomatic manifestations (dyspnea and fatigue), echocardiographic evidence of impaired cardiac function (left ventricular ejection fraction [EF] <40%), and relevant medical history. Inclusion criteria were: age 45–75 years; HF diagnosis according to the 2022 ESC Guidelines for the Diagnosis and Treatment of Acute and Chronic HF; and willingness to provide informed consent. Exclusion criteria included severe hepatic or renal dysfunction, history of malignant tumors, and unwillingness to participate.

The study protocol was approved by the Institutional Ethics Committee of the Affiliated Hospital of Inner Mongolia Medical University, and written informed consent was obtained from all participants prior to sample collection.

A control group comprising five age‐ and gender‐matched healthy individuals without cardiovascular disease, HF symptoms, or other major chronic illnesses was concurrently recruited. Written informed consent was obtained from all healthy volunteers. Routine physical examinations and basic laboratory tests confirmed their eligibility as controls.

Blood samples were collected from both HF patients and healthy controls under standardized procedures for subsequent plasma exosome isolation and analysis.

### 2.3 Exosome isolation and purification

Collected blood samples were first centrifuged at 1500 × *g* for 10 min at 4°C to remove blood cells. The resulting supernatant was carefully transferred to new tubes and further centrifuged at 3000 × *g* for 15 min at 4°C to eliminate residual cell debris. The supernatant obtained after this step constituted plasma, which was subsequently centrifuged to pellet large vesicles. The clarified plasma was then transferred to ultracentrifuge tubes and subjected to ultracentrifugation at 100,000 × *g* for 70 min at 4°C. The exosome‐containing pellet was resuspended in an appropriate volume of phosphate‐buffered saline (PBS) for downstream experiments.

### 2.4 Exosome uptake assay

For exosome uptake assays, purified exosomes were labeled with PKH26 and incubated with AC16 cells (1 × 10^5^ cells/well) for 4 h. Following incubation, cells were washed with PBS, fixed in 4% paraformaldehyde for 15 min, washed again, and examined under an inverted fluorescence microscope (Shanghai Yiyuan Optical Instrument Co., Ltd.).

For functional exosome treatments, AC16 cells were pre‐treated with Ang II (1 μM) for 24 h and subsequently incubated with circulating exosomes derived from healthy controls (NC‐exo) or HF patients (HF‐exo) for an additional 24 h. Exosomal miR‐339‐3p uptake was evaluated by quantifying intracellular miR‐339‐3p levels via reverse transcription‐quantitative polymerase chain reaction (RT‐qPCR).

### 2.5 Exosome identification

#### 2.5.1 Verification of exosome markers by Western blot

Protein concentration was determined using a bicinchoninic acid (BCA) protein assay kit (Beyotime Biotechnology). Equal amounts of protein were resolved by 10% sodium dodecyl sulfate‐polyacrylamide gel electrophoresis (SDS‐PAGE) and transferred onto polyvinylidene difluoride (PVDF) membranes. Membranes were blocked with 5% non‐fat milk for 1 h at room temperature and incubated overnight at 4°C with primary antibodies against exosomal markers: CD9 (1:1,000, Cell Signaling Technology), CD63 (1:1,000, Cell Signaling Technology), TSG101 (1:1,000, Santa Cruz Biotechnology), and GAPDH (1:5,000, Cell Signaling Technology). After washing with Tris‐Buffered Saline with Tween‐20, membranes were incubated with HRP‐conjugated secondary antibodies (1:5000) for 2 h at room temperature. Protein bands were visualized using an enhanced chemiluminescence (ECL) detection system (Thermo Fisher Scientific) and analyzed quantitatively for marker expression.

#### 2.5.2 Observation of exosome morphology by transmission electron microscope

Exosome morphology was examined by a transmission electron microscope (TEM). Briefly, a 3‐μL aliquot of resuspended exosome pellet was placed onto a 200‐mesh copper grid coated with Formvar–carbon film and incubated at room temperature for 5 min. Grids were washed with PBS, stained with 2% uranyl acetate, rinsed with PBS, semi‐dried at room temperature, and observed under TEM (Thermo Fisher Scientific Co., Ltd.).

### 2.6 Cell counting kit‐8 assay for cell viability

Cell viability was assessed using a cell counting kit‐8 (CCK‐8) assay. After 48 h of Ang II treatment, 10 μL of CCK‐8 reagent (Beyotime Biotechnology) was added to each well of a 96‐well plate containing 100 μL of treated AC16 cells per well. Plates were incubated at 37°C for 4 h, after which absorbance at 450 nm was measured using a microplate reader (Bio‐Rad). Cell viability was calculated as a percentage relative to the control group using the formula: cell viability (%) = (absorbance of treatment group/absorbance of NC group) × 100%.

### 2.7 Flow cytometry analysis for cell apoptosis

AC16 cells were harvested and washed twice with cold PBS. Subsequently, the cells were resuspended in 100 μL of binding buffer containing Annexin V‐FITC and propidium iodide (PI), according to the manufacturer's instructions of the Annexin V‐FITC/PI apoptosis detection kit.^15^ The cell suspensions were incubated in the dark at room temperature for 15 min. Thereafter, 400 μL of binding buffer was added to each sample, and apoptosis was quantified by flow cytometry (BD Biosciences).

### 2.8 Terminal deoxynucleotidyl transferase dUTP nick end labeling (TUNEL) staining for cardiomyocyte apoptosis

Cardiomyocyte apoptosis was further assessed via TUNEL staining using a TUNEL apoptosis detection kit (Beyotime Biotechnology) following the manufacturer's protocol. AC16 cells or mouse heart tissue sections were washed three times with PBS, fixed in 4% paraformaldehyde for 30 min, and permeabilized with 0.1% Triton X‐100 for 10 min at room temperature. Samples were blocked with 10% normal goat serum for 15 min and then incubated with the TUNEL reaction mixture at 37°C in the dark for 1 h. Nuclear counterstaining was performed with 4', 6'‐diamidino‐2‐phenylindole (DAPI) for 5 min. The stained samples were observed under a fluorescence microscope (Shanghai Yiyuan Optical Instrument Co., Ltd.), and the apoptotic rate was quantified using ImageJ software (National Institutes of Health).

### 2.9 WGA staining for cardiomyocyte cross‐sectional area

Wheat germ agglutinin (WGA) staining was used to determine cardiomyocyte cross‐sectional area. Following treatment, AC16 cells or mouse heart tissue sections were washed three times with PBS, fixed in 4% paraformaldehyde for 30 min, and permeabilized with 0.1% Triton X‐100 for 10 min. Samples were incubated with Alexa Fluor‐conjugated WGA (1:200, Thermo Fisher Scientific) at 37°C for 30 min in the dark. DAPI was used for nuclear counterstaining. Fluorescence images were captured, and cardiomyocyte cross‐sectional area was quantified using ImageJ software.

### 2.10 FITC‐phalloidin staining for surface area of AC16 cells

For assessment of cardiomyocyte surface area, FITC‐phalloidin staining was employed. Post‐treatment, AC16 cells were washed three times with PBS, fixed with 4% paraformaldehyde for 30 min, and permeabilized with 0.1% Triton X‐100 for 10 min. Cells were blocked with 10% normal goat serum for 10 min and incubated with FITC‐phalloidin (Sigma‐Aldrich) at 37°C for 30 min. Stained cells were visualized under a fluorescence microscope (Shanghai Yiyuan Optical Instrument Co., Ltd.), and the surface area was quantified using ImageJ software (National Institutes of Health).

### 2.11 Ribonucleic acid (RNA) extraction and reverse transcription‐quantitative polymerase chain reaction assays

To investigate the effects of plasma exosomes on cardiomyocyte apoptosis and hypertrophy, as well as the expression of miR‐339‐3p and mRNAs of ANP, BNP, Myh7, USP25, and DDX58, total RNA was extracted from AC16 cells and tissue samples using TRIzol reagent. Primers used in this assay are listed in Table 1. Briefly, cells were lysed directly in culture wells with TRIzol reagent, followed by addition of chloroform and centrifugation to separate the aqueous phase. The upper aqueous phase containing RNA was collected, and RNA was precipitated with isopropanol. The RNA pellet was washed with 75% ethanol, air‐dried, and resuspended in RNase‐free water. RNA concentration and purity were measured using a spectrophotometer.

**TABLE 1:** Primer sequences used in this study.

Name	Sequence
ANP‐forward	5′‐AGTCATCTACACTGGAGTCC‐3′
ANP‐reverse	5′‐AGTGTTGATTCCTTCACCAC‐3′
BNP‐forward	5′‐AACAGCGACGCTGTGGTCAG‐3′
BNP‐reverse	5′‐GACTCGAACGATGGTTTTAAC‐3′
Myh7‐forward	5′‐CCTAGCATCTCAGGCATCTGGGTCGTGGAGTG‐3′
Myh7‐reverse	5′‐CCTTGGCAGAAACCCTGCTCCTCTGTACCG‐3′
USP25‐forward	5′‐GCCATGACCGTGGAGCAGAACG‐3′
USP25‐reverse	5′‐CAGCACTAAACCAACAAGTATTGCCA‐3′

Abbreviations: ANP, A‐type natriuretic peptide; BNP, B‐type natriuretic peptide; USP25, ubiquitin‐specific protease 25.

Reverse transcription was performed to synthesize cDNA from extracted RNA. Subsequently, RT‐qPCR was carried out using gene‐specific primers for ANP, BNP, and Myh7. Relative expression levels were quantified by analyzing real‐time fluorescence signals.

### 2.12 Western blot analysis

Protein lysates from AC16 cells or mouse heart tissues were prepared using radio‐immunoprecipitation assay lysis buffer (Thermo Fisher Scientific). Protein concentration was quantified using a BCA protein assay kit (Beyotime Biotechnology), and equal amounts of protein were loaded for subsequent analyses. The procedures for SDS‐PAGE, transfer to PVDF membranes, blocking, and antibody incubation followed those described in Section 2.5.1. Primary antibodies specific for ANP (1:1000, Thermo Fisher Scientific), BNP (1:1000, Thermo Fisher Scientific), Myh7 (1:1000, Cell Signaling Technology), USP25 (1:1000, Cell Signaling Technology), DDX58 (1:1000, Cell Signaling Technology), and GAPDH (1:5000, Cell Signaling Technology) were incubated overnight at 4°C. Membranes were subsequently washed and incubated with horseradish peroxidase‐conjugated secondary antibodies at room temperature for 2 h. Protein bands were visualized using an ECL detection system (Thermo Fisher Scientific).

### 2.13 Screening of microRNA (miRNA) and downstream target gene

Differentially expressed miRNAs were screened via next‐generation sequencing. Following reverse transcription and library construction, sequencing data were processed, and differentially expressed miRNAs were identified using DESeq2, with criteria set as |log_2_(fold change)| > 1.0 and adjusted *p* value < 0.05. Volcano plots were generated, with log_2_(fold change) on the *x*‐axis and −log_10_(adjusted *p* value) on the *y*‐axis, to visualize significant miRNA expression changes. Heatmaps were constructed for a subset of significantly differentially expressed miRNAs, displaying relative expression patterns across samples.

To predict downstream target genes of the key miRNA (miR‐339‐3p), cross‐validation was performed using TargetScan (https://www.targetscan.org/vert_80/), miRDB (http://mirdb.org/), and Starbase (http://starbase.sysu.edu.cn/).

### 2.14 Functional and pathway clustering

Functional enrichment analyses of differentially expressed genes were conducted using gene ontology (GO) terms and Kyoto encyclopedia of genes and genomes (KEGG) pathways, with a corrected *p* < 0.05 set as the threshold for significant enrichment. Enrichment factors were also calculated to reflect the degree of gene‐term association.

### 2.15 Luciferase reporter assay

For luciferase reporter assays, wild‐type (WT) or mutant USP25 3' untranslated region (3′UTR) plasmids were co‐transfected with miR‐339‐3p mimics into AC16 cells using Lipofectamine 2000. After 24 h of incubation, cells were lysed, and dual‐luciferase reporter activity was measured using a dual‐luciferase assay kit (Thermo Fisher Scientific) according to the manufacturer's instructions.

### 2.16 Co‐immunoprecipitation and ubiquitination assay

To investigate the interaction between USP25 and DDX58 and assess ubiquitination modification, AC16 cells were co‐transfected with HA‐USP25 and flag‐DDX58 plasmids and cultured for 24 h. The medium was then replaced with medium containing 25 μM MG132 (Omer Biotechnology) for an additional 6 h. Cells were lysed in 0.6 mL lysis buffer (Beyotime) for 45 min, followed by centrifugation at 12,500 × *g* for 15 min. Supernatants were incubated overnight at 4°C with 1.2 μg of antibodies against USP25 or DDX58. Protein A/G agarose beads (35 μL, Invitrogen) were washed and added to the antigen‐antibody complexes for 3 h of incubation. Finally, the bead‐antigen‐antibody complexes were boiled with loading buffer for 4 min and subjected to SDS‐PAGE and Western blot analysis.

### 2.17 Animal model

Male C57BL/6 mice (8–10 weeks old, *n* = 32, mean body weight 25 g) were purchased from Shanghai Laboratory Animal Center, Chinese Academy of Sciences, and housed under controlled conditions (24°C, 12 h light/dark cycle). All experimental procedures were performed in accordance with the guidelines for the care and use of laboratory animals established by the Affiliated Hospital of Inner Mongolia Medical University.

Pathological cardiac remodeling mimicking HF was induced via transverse aortic constriction (TAC) surgery using an optimized protocol.^12^ Mice were anesthetized by intraperitoneal injection of pentobarbital sodium (50 mg/kg) and positioned supine. A midline incision exposed the sternum, which was incised at the level of the second rib. Thymic tissue was retracted to visualize the aortic arch and carotid arteries. A 27‐gauge needle was placed parallel to the transverse aorta as a reference, and the aorta was ligated using a 6‐0 nylon suture. The chest was subsequently closed in layers. Sham‐operated controls underwent identical surgical exposure without ligation and served as NCs.

### 2.18 Transfection of interference and overexpression vectors

To achieve in vivo modulation of target gene expression, lentiviral vectors encoding shRNA (VectorBuilder) or overexpression (OE) plasmids were constructed. USP25 OE plasmids (Qinsheng Bio) were used to upregulate target gene expression, with empty pcDNA3.1 vectors (Thermo Fisher Scientific) serving as NCs. For miR‐339‐3p knockdown, sh‐miR‐339‐3p lentiviral vectors (Thermo Fisher Scientific) and control sh‐NC were employed. Mice were intravenously injected with lentivirus (1 × 10^9^ TU/mL, 200 μL per mouse) or plasmid vectors (100 μg per mouse) at the beginning of the experiment, with weekly repeat injections.

### 2.19 Histological assessment of myocardial degeneration by hematoxylin‐eosin staining

After 4 weeks of treatment, mouse hearts were collected for histological assessment. Hearts were fixed in 4% paraformaldehyde, dehydrated through graded ethanol, cleared with xylene, and embedded in paraffin. Serial sections (4–5 μm) were deparaffinized, rehydrated, and stained with hematoxylin and eosin (HE, Beijing Solarbio Science & Technology Co.). Following dehydration, clearing, and mounting, the sections were examined under a light microscope (Leica Microsystems, Germany) to evaluate myocardial structural changes.

### 2.20 Assessment of cardiac function and physiological parameters

For in vivo miR‐339‐3p inhibition, anti‐miR‐339‐3p or control antagomir (5 mg/kg) was administered via tail vein weekly. After 4 weeks, heart weight/tibial length (HW/TL) ratios were calculated. Cardiac function was assessed by transthoracic echocardiography. Mice were anesthetized with 2% isoflurane (Sinopharm Chemical Reagent), chest hair was removed, and a small amount of ultrasonic coupling gel was applied. A 30 MHz linear array probe was used to measure left ventricular EF and fractional shortening.

### 2.21 Statistical analysis

Data are expressed as mean ± standard deviation. Comparisons between two groups were performed using Student's *t*‐test. For comparisons involving three or more groups, one‐way analysis of variance (ANOVA) followed by Tukey's post hoc test was applied. All statistical analyses were conducted using GraphPad Prism 8. For experiments involving two independent factors, two‐way ANOVA was conducted, followed by Tukey's post hoc test to evaluate main effects and interaction effects. Correlations between USP25 and DDX58 expression were analyzed using Pearson correlation analysis. *p* < 0.05 was considered statistically significant.

## 3 RESULTS

### 3.1 Identification of exosome

**FIGURE 1:**
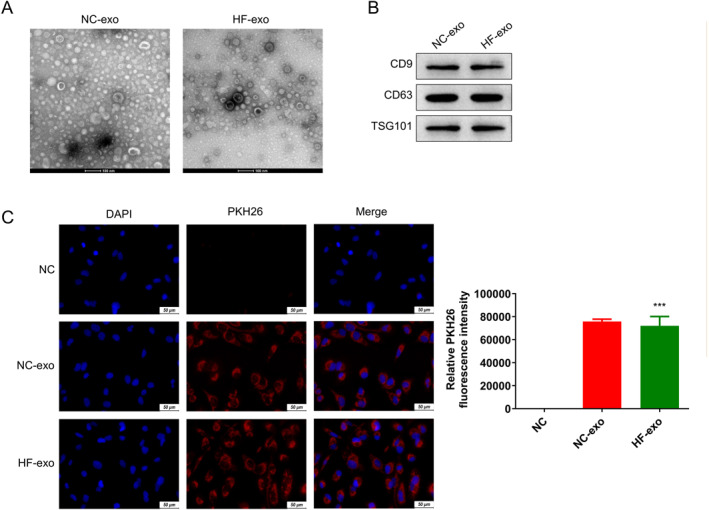
Exosomes isolated from the healthy controls and HF patients. To confirm the extraction efficiency of plasma exosomes from patients with HF and their uptake ability by cardiomyocytes, plasma samples from 5 HF patients and 5 healthy individuals were used as research objects. (A) Transmission electron microscopy images of exosomes from healthy controls and HF patients, displaying round or cup‐shaped vesicles with a diameter of 30–100 nm, which is consistent with the typical morphological characteristics of exosomes. (B) The expression of exosomal markers (CD9, CD63 and TSG101) by western blot analysis. (C) Confocal microscopy showing PKH26‐labeled exosomes were taken up by AC16 cardiomyocytes. HF, heart failure.

In this study, blood samples were collected from five patients with HF and five age‐ and gender‐matched healthy controls, and plasma exosomes were isolated via ultracentrifugation. TEM revealed that the isolated vesicles exhibited a round morphology with diameters ranging from 30 to 100 nm (Figure 1A), consistent with the characteristic structure of exosomes.^16^ Western blot analysis confirmed the presence of exosomal markers CD9, CD63, and TSG101 (Figure 1B).^17^ Moreover, exosomes labeled with the red fluorescent dye PKH‐26 were internalized by AC16 cardiomyocytes following co‐incubation (Figure 1C), demonstrating the successful uptake of plasma‐derived exosomes by cardiomyocytes. These results confirmed the successful isolation of functional exosomes from plasma.

### 3.2 Plasma exosomes from HF patients promote cardiomyocyte apoptosis and hypertrophy

**FIGURE 2:**
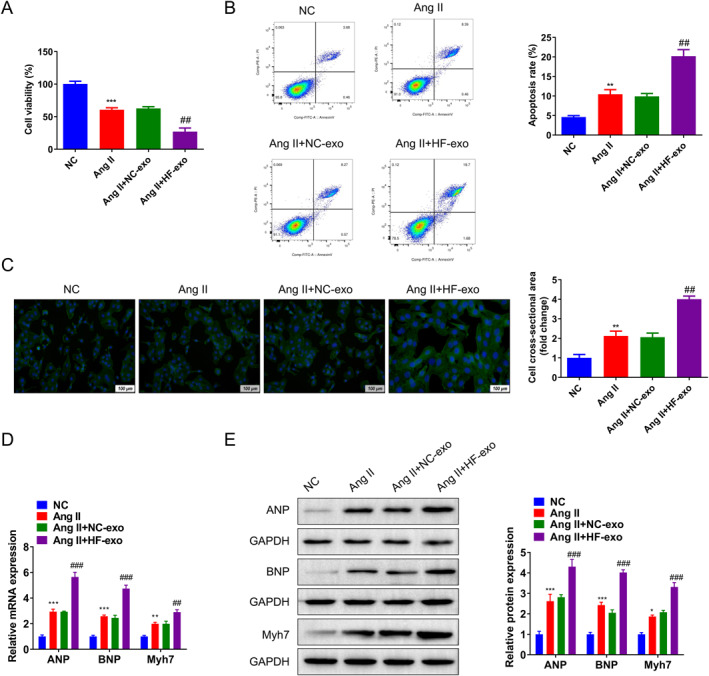
Plasma exosomes from HF patients promote cardiomyocyte apoptosis and hypertrophy. To investigate the effect of plasma exosomes from HF patients on cardiomyocytes, AC16 cells were divided into four groups: normal control group (NC), angiotensin II‐treated group (Ang II), Ang II + healthy human plasma exosome group (Ang II + NC‐exo), and Ang II + HF‐exo group. (A) CCK‐8 assay for detecting cell viability, showing that compared with the NC group, cell viability in the Ang II group is significantly reduced, and the Ang II + HF‐exo group exhibits a further decrease in cell viability compared to the Ang II and Ang II + NC‐exo groups. (B) Flow‐cytometry for detecting apoptosis, revealing that the apoptosis rate in the Ang II group is higher than that in the NC group, and the Ang II + HF‐exo group has a significantly higher apoptosis rate than the Ang II and Ang II + NC‐exo groups. (C) The surface area of AC16 cells after FITC‐Phalloidin staining, showing that the Ang II group has a significantly increased hypertrophy area compared to the NC group. (D, E) RT‐PCR and Western blot for detecting the expression of hypertrophy‐related markers A‐type natriuretic peptide, B‐type natriuretic peptide, and Myh7, demonstrating that their expression in the Ang II group is higher than that in the NC group, and the Ang II + HF‐exo group shows significantly higher expression than the Ang II and Ang II + NC‐exo groups. *, **, and *** represent *p* < 0.05, *p* < 0.01, and *p* < 0.001 compared to the NC group. ^##^, and ^###^ represents *p* < 0.01, and *p* < 0.001 compared to the Ang II group. HF, heart failure; NC, negative control.

To investigate the effect of HF‐derived plasma exosomes (HF‐exo) on cardiomyocytes, AC16 cells were first treated with Ang II to induce a hypertrophic phenotype, followed by exosome treatment. Four experimental groups were established: NC group, Ang II group, Ang II + NC‐exo group, and Ang II + HF‐exo group. Compared with the NC group, Ang II treatment alone significantly reduced cardiomyocyte viability (CCK‐8 assay, Figure 2A), increased apoptosis (flow cytometry, Figure 2B), enlarged cell surface area (FITC‐phalloidin staining, Figure 2C), and upregulated the expression of pathological hypertrophy markers ANP, BNP, and Myh7 at both the mRNA level (RT‐qPCR, Figure 2D) and protein level (Western blot, Figure 2E). Co‐treatment with NC‐exo did not alter these phenotypic changes. In contrast, AC16 cells treated with both Ang II and HF‐exo exhibited a marked exacerbation of all pathological features: Cell viability was further reduced, apoptosis rates were increased, hypertrophy was more severe, and the expression of ANP, BNP, and Myh7 was significantly upregulated compared with both the Ang II and Ang II + NC‐exo groups. These results indicate that exosomes derived from HF patients exacerbate Ang II‐induced cardiomyocyte dysfunction, suggesting that HF plasma exosomes carry pro‐hypertrophic and pro‐apoptotic factors.

### 3.3 Identification of miR‐339‐3p as a key differential miRNA in HF exosomes

**FIGURE 3:**
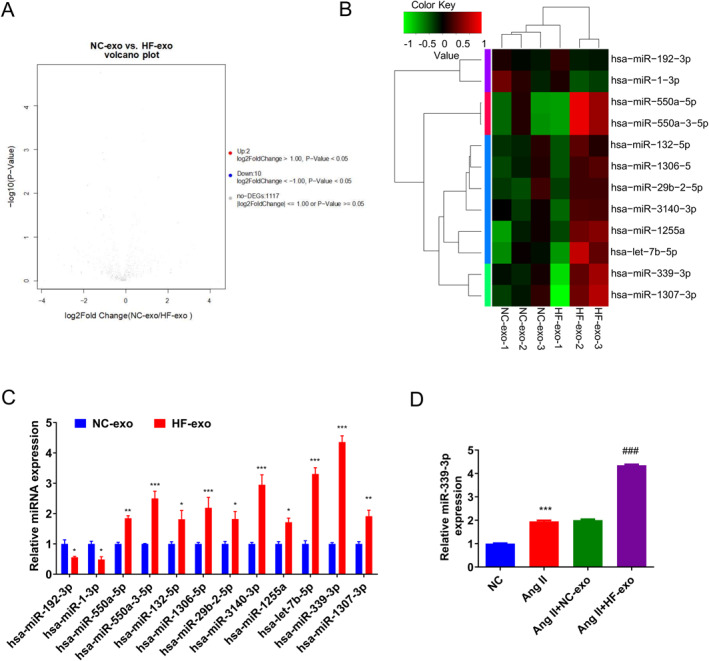
Screening of different expressed blood exosomes miRNA between healthy controls and HF patients. To screen for differentially expressed miRNAs in plasma exosomes from HF patients (HF‐exo) and healthy individuals (NC‐exo), miRNA sequencing analysis was performed on the two groups of exosomes. A total of 1129 miRNAs were detected. (A) Volcano plot analysis of miRNA expression differences, identifying 12 differentially expressed miRNAs. (B) Heatmap analysis displays the expression patterns of differential miRNAs in NC‐exo and HF‐exo samples. (C) Reverse transcription‐quantitative polymerase chain reaction validation of differently expressed miRNAs. (D) HF patient‐derived exosomes increase miR‐339‐3p expression in Ang II‐treated cardiomyocytes. *, **, and *** represent *p* < 0.05, *p* < 0.01, and *p* < 0.001 compared to the NC‐exo group, respectively. ^###^ *p* < 0.0001 versus Ang II group. HF, heart failure; miRNA, microRNA; NC, negative control.

To identify differentially expressed miRNAs in plasma exosomes between HF patients and healthy controls, miRNA sequencing was performed, detecting a total of 1129 miRNAs (Figure 3A). Volcano plot and heatmap analyses indicated that 12 miRNAs were significantly differentially expressed (*p* < 0.05, |log2FoldChange| > 1.0), of which 2 were upregulated and 10 were downregulated (Figure 3A,B). The remaining 1117 miRNAs showed no significant differences. To validate and prioritize the most significantly altered miRNA, RT‐qPCR was conducted on plasma exosomes from both groups, identifying miR‐339‐3p as the most prominently differentially expressed miRNA (Figure 3C). As shown in Figure 3D, Ang II treatment alone significantly increased endogenous miR‐339‐3p expression in AC16 cells (**p < 0.001 vs. NC). Importantly, co‐treatment with HF patient‐derived exosomes further markedly elevated miR‐339‐3p levels in Ang II‐stimulated cardiomyocytes (^####^ *p* < 0.0001 vs. Ang II group), whereas exosomes from healthy controls (NC‐exo) did not produce an additional increase. These results indicate that circulating exosomes from HF patients deliver exogenous miR‐339‐3p into cardiomyocytes, effectively increasing intracellular levels.

GO analysis (Figure S1A) demonstrated that the differentially expressed miRNAs were predominantly enriched in biological processes such as regulation of gene expression, cellular protein modification, and regulation of metabolic processes. KEGG pathway analysis (Figure S1B) revealed enrichment in pathways including “MicroRNAs in cancer” and the “MAPK signaling pathway.” These results suggest that exosomal miRNAs may modulate HF progression by regulating these biological processes and signaling pathways.

### 3.4 miR‐339‐3p in HF‐exo drives cardiomyocyte apoptosis and hypertrophy

**FIGURE 4:**
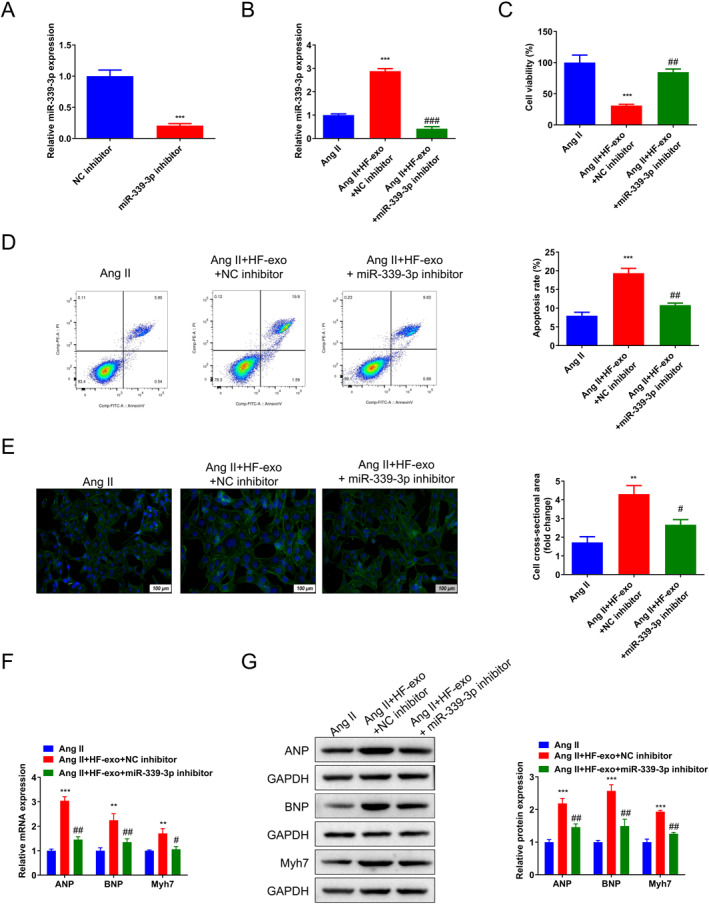
miR‐339‐3p in plasma exosomes of HF patients promotes cardiomyocyte apoptosis and hypertrophy. To verify the regulatory role of miR‐339‐3p in plasma exosomes from HF patients on cardiomyocytes, AC16 cells were divided into three groups: Ang II group, Ang II + HF‐exo + negative control inhibitor group (Ang II + HF‐exo + NC inhibitor), and Ang II + HF‐exo + miR‐339‐3p inhibitor group (Ang II + HF‐exo + miR‐339‐3p inhibitor). (A) Transfection efficiency detected by RT‐qPCR, confirming that the miR‐339‐3p inhibitor can effectively reduce the expression of miR‐339‐3p in cells. (B) Expression of miR‐339‐3p in AC16 cells detected by RT‐qPCR. (C) Cell viability detected by CCK‐8 assay shows that compared with the Ang II and Ang II + HF‐exo + NC inhibitor groups, the Ang II + HF‐exo + miR‐339‐3p inhibitor group has significantly increased cell viability. (D) Cell death detected by flow cytometry. (E) FITC‐phalloidin staining of surface area of AC16 cells. (F) Expression of pathological hypertrophy markers ANP, BNP, and Myh7 detected by RT‐qPCR. (G) Expression of pathological hypertrophy markers ANP, BNP and Myh7 detected by western blot analysis. ** and *** represent *p* < 0.01 and *p* < 0.001 compared to the control group (NC inhibitor group or Ang II group). ^#^, ^##^, and ^###^ represents *p* < 0.05, *p* < 0.01, and *p* < 0.001 compared to the Ang II + HF‐exo + NC inhibitor group. ANP, A‐type natriuretic peptide; BNP, B‐type natriuretic peptide; HF, heart failure; NC, negative control; RT‐qPCR, reverse transcription‐quantitative polymerase chain reaction.

To confirm the functional role of miR‐339‐3p in cardiac hypertrophy, a specific miR‐339‐3p inhibitor was employed to silence its expression. Accordingly, three experimental groups were established: Ang II, Ang II + HF‐exo + NC inhibitor, and Ang II + HF‐exo + miR‐339‐3p inhibitor. RT‐qPCR results demonstrated that treatment with the miR‐339‐3p inhibitor significantly reduced miR‐339‐3p expression, confirming successful knockdown in AC16 cells (Figure 4A,B). Compared with the Ang II + HF‐exo + NC inhibitor group, functional assays revealed that inhibition of miR‐339‐3p markedly increased cardiomyocyte viability (CCK‐8 assay, Figure 4C), decreased apoptosis rate (flow cytometry, Figure 4D), reduced cell surface area (FITC‐Phalloidin staining, Figure 4E), and downregulated the expression of hypertrophic markers ANP, BNP, and Myh7 at both the mRNA (RT‐qPCR, Figure 4F) and protein levels (Western blot, Figure 4G). These results indicate that miR‐339‐3p carried by HF‐exo acts as a critical mediator of the observed pathological effects.

To further validate these results in a clinically relevant context, key assays were replicated using hiPSC‐derived cardiomyocytes (hiPSC‐CMs). The transfection efficiency of the miR‐339‐3p inhibitor was confirmed (Figure S2A). In Ang II‐stimulated hiPSC‐CMs, HF‐exo treatment significantly increased miR‐339‐3p expression, and this effect was effectively blocked by miR‐339‐3p inhibition (Figure S2B). Functionally, HF‐exo exacerbated Ang II‐induced reductions in cell viability (Figure S2C), increased apoptosis (Figure S2D), enlarged cell cross‐sectional area (Figure S2E), and upregulated hypertrophic markers (ANP, BNP, Myh7) at both the mRNA (Figure S2F) and protein levels (Figure S2G). All these detrimental effects were significantly attenuated by miR‐339‐3p inhibition. Collectively, these results demonstrate that silencing miR‐339‐3p mitigates the pro‐apoptotic and pro‐hypertrophic effects of HF patient‐derived exosomes in hiPSC‐CMs, reinforcing the conclusion that exosomal miR‐339‐3p contributes to myocardial remodeling in HF.

### 3.5 Screening of miR‐339‐3p target genes

**FIGURE 5:**
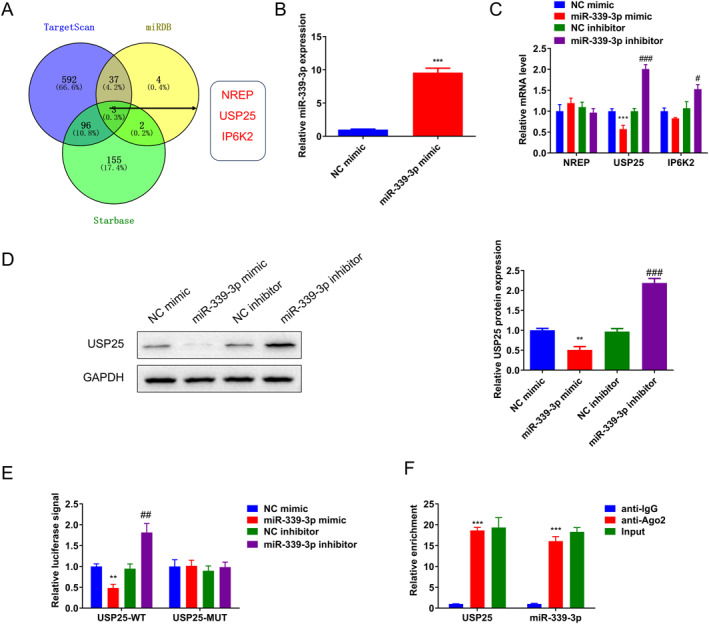
Identification of downstream target genes of miR‐339‐3p. (A) To screen the downstream target genes of miR‐339‐3p, predictive analysis was conducted using three databases: TargetScan (728 target genes), miRDB (46 target genes), and Starbase (256 target genes). (B) RT‐qPCR detection of miR‐339‐3p expression in cells after transfection with miR‐339‐3p mimic. (C) In order to explore the effect of miR‐339‐3p on NREP, USP25 and IP6K2 genes, we set up four treated groups: the NC mimic group, miR‐339‐3p mimic group, NC inhibitor group and miR‐339‐3p inhibitor group. Expression of target genes NREP, USP25 and IP6K2 in AC16 cells detected by RT‐qPCR analysis. (D) Expression of target gene USP25 in different treated groups accessed by western blot analysis. (E) Luciferase assay used to confirm the miR‐339‐3p targets USP25. (F) RNA immunoprecipitation assay performed to confirm that miR‐339‐3p targets USP25. ** and *** represent *p* < 0.01 and *p* < 0.001 compared to the control group (NC mimic group or anti‐IgG group). ^#^ and ^###^ represents *p* < 0.05 and *p* < 0.001 compared to the miR‐339‐3p mimic group or NC inhibitor group. NC, negative control; RNA, Ribonucleic acid; RT‐qPCR, reverse transcription‐quantitative polymerase chain reaction; USP25, ubiquitin‐specific protease 25.

To investigate the molecular mechanism underlying the effects of exosomal miR‐339‐3p, potential target genes were predicted using TargetScan, miRDB, and Starbase databases. Three candidate genes—NREP, USP25, and IP6K2—were identified (Figure 5A). To validate the regulatory relationship between miR‐339‐3p and these targets, AC16 cells were transfected with a miR‐339‐3p mimic. RT‐qPCR confirmed a significant increase in miR‐339‐3p levels compared with the NC mimic group, indicating successful transfection (Figure 5B). Subsequent analysis of the mRNA levels of the candidate genes revealed that USP25 was downregulated in the miR‐339‐3p mimic group and upregulated in the miR‐339‐3p inhibitor group (Figure 5C). This study further investigated the protein level of USP25 using western blot analysis, and the results were consistent with the mRNA results, showing that USP25 was significantly downregulated in the miR‐339‐3p mimic group and upregulated in the miR‐339‐3p inhibitor group (Figure 5D). To determine whether miR‐339‐3p directly targets USP25, a dual‐luciferase reporter assay was performed (Figure 5E). The results demonstrated that the miR‐339‐3p mimic significantly reduced luciferase activity of the WT USP25 3′UTR reporter plasmid (USP25‐WT), whereas the miR‐339‐3p inhibitor increased luciferase activity of USP25‐WT. In contrast, neither mimic nor inhibitor affected the mutant USP25 reporter (USP25‐Mut) (Figure 5E). Finally, RNA immunoprecipitation assays confirmed the interaction between miR‐339‐3p and USP25 within the RNA‐induced silencing complex. Both USP25 and miR‐339‐3p were significantly enriched in the Ago2 immunoprecipitate relative to IgG controls (Figure 5F), verifying their association. These results confirm that miR‐339‐3p directly targets the 3′UTR of USP25.

### 3.6 miR‐339‐3p promotes cardiomyocyte dysfunction by targeting and inhibiting USP25

**FIGURE 6:**
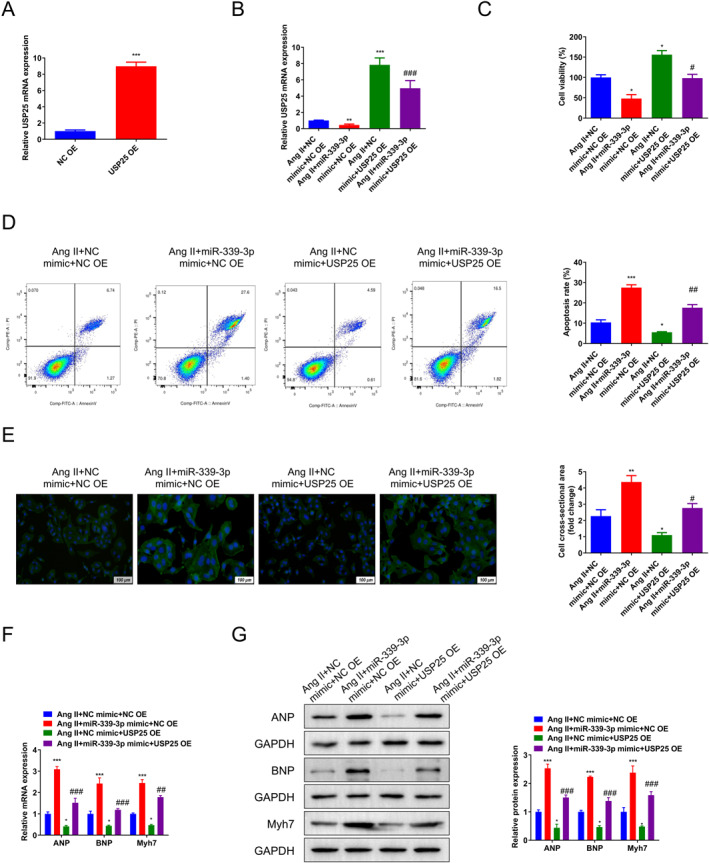
miR‐339‐3p promotes cardiomyocyte apoptosis and hypertrophy by targeting USP25. To verify whether miR‐339‐3p regulates cardiomyocyte function by targeting USP25, AC16 cells were divided into four groups: Ang II + NC mimic + NC OE group (Ang II + NC mimic + NC OE), Ang II + miR‐339‐3p mimic + NC OE group (Ang II + miR‐339‐3p mimic + NC OE), Ang II + NC mimic + USP25 OE group (Ang II + NC mimic + USP25 OE), and (Ang II + miR‐339‐3p mimic + USP25 OE). (A) Transfection efficiency of USP25 OE. (B) RT‐qPCR results of USP25 relative mRNA expression. (C) Cell viability detected by CCK‐8. (D) Detection of cell apoptosis by flow cytometry. (E) Detection of cardiomyocyte volume by immunofluorescence staining. (F) Detection of pathological hypertrophy markers ANP, BNP, and Myh7 expression by RT‐qPCR. (G) Expression of pathological hypertrophy markers ANP, BNP, and Myh7 detected by western blot analysis. *, **, and *** represent *p* < 0.05, *p* < 0.01, and *p* < 0.001 compared to the control group (Ang II + NC mimic + NC OE group). ^#^, ^##^, and ^###^ represents *p* < 0.05, *p* < 0.01, and *p* < 0.001 compared to the Ang II + NC mimic + USP25 OE group. ANP, A‐type natriuretic peptide; BNP, B‐type natriuretic peptide; NC, negative control; OE, overexpression; RT‐qPCR, reverse transcription‐quantitative polymerase chain reaction; USP25, ubiquitin‐specific protease 25.

To verify the regulatory relationship and functional significance between miR‐339‐3p and USP25, a rescue experiment was conducted in AC16 cells. Cells were first transfected with a miR‐339‐3p mimic, followed by co‐transfection with a USP25 OE vector. RT‐qPCR analysis demonstrated that USP25 mRNA levels were markedly elevated in the USP25 OE‐treated group, confirming successful OE (Figure 6A). Subsequently, the effect of miR‐339‐3p on USP25 expression was examined. Compared with the Ang II + NC mimic + NC OE group, OE of miR‐339‐3p significantly downregulated USP25 mRNA levels, whereas co‐transfection with the USP25 OE vector partially restored USP25 expression, supporting that miR‐339‐3p negatively regulates USP25 at the mRNA level (Figure 6B). Functional assays, including CCK‐8, flow cytometry, immunofluorescence staining, RT‐qPCR, and Western blot, were performed to evaluate the effects of miR‐339‐3p and USP25 on cardiomyocyte function. OE of miR‐339‐3p significantly reduced cell viability (Figure 6C), increased apoptosis (Figure 6D), and enlarged the cardiomyocyte cross‐sectional area (Figure 6E) relative to the Ang II‐treated control group. Notably, USP25 OE partially mitigated these adverse effects. Furthermore, the expression levels of cardiac hypertrophy markers (ANP, BNP, and Myh7) were assessed by RT‐qPCR and Western blot (Figure 6F,G). Results indicated that miR‐339‐3p OE increased both mRNA and protein levels of these markers, reflecting induction of hypertrophy‐like changes, whereas USP25 OE reversed these changes, suggesting that miR‐339‐3p mediates its effects on cardiomyocyte function through USP25 targeting.

### 3.7 USP25 regulates the translation of DDX58 by mediating its deubiquitination

**FIGURE 7:**
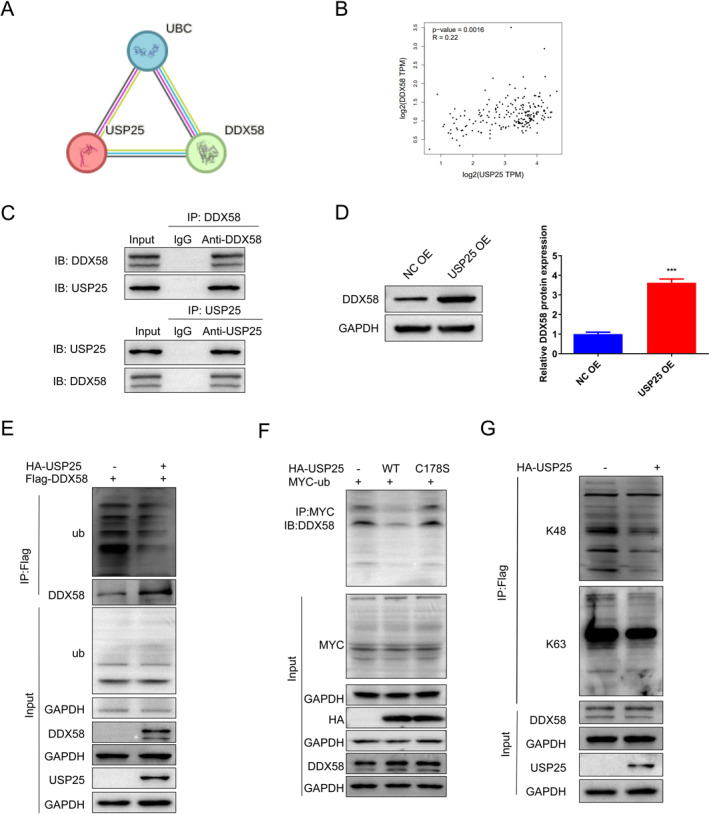
USP25 promotes the deubiquitination of DDX58. To explore the interaction between USP25 and DDX58 and its regulatory effect on DDX58 ubiquitination, a series of experiments were conducted. (A) Protein–protein interaction prediction analysis on USP25 using the String website detects DDX58 as an interacting protein of UPS25. (B) Pearson correlation analysis based on the TCGA database shows a positive correlation between USP25 and DDX58 expression in human left ventricular tissues. (C) Relationship between USP25 and DDX58 detected by co‐immunoprecipitation analysis. (D) Expression of DDX58 in negative control OE group and USP25 OE group detected by western blot analysis. (E) Ubiquitination level of DDX58 detected by IP experiment with overexpressed USP25. (F) Endogenous DDX58 ubiquitination level detected by IP with MYC‐tagged antibody. (G) K48 and K63 ubiquitin chains of DDX58 detected by western blot analysis. OE, overexpression; TCGA, the Cancer Genome Atlas; USP25, ubiquitin‐specific protease 25.

To explore the downstream mechanisms of USP25, protein–protein interactions were analyzed using the search tool for the retrieval of interacting genes (STRING) database. DDX58 was identified as a potential binding partner (Figure 7A). Analysis of the Cancer Genome Atlas datasets revealed a positive correlation between USP25 and DDX58 expression in human left ventricular tissues (Figure 7B). Co‐immunoprecipitation (Co‐IP) experiments in AC16 cells confirmed a physical interaction between USP25 and DDX58 (Figure 7C). Western blot analysis demonstrated that USP25 OE increased DDX58 protein levels (Figure 7D). Ubiquitination assays further revealed that USP25 OE reduced DDX58 ubiquitination (Figure 7E), with WT USP25 exerting a stronger effect than the enzymatic mutant C178S (Figure 7F). Additional assays showed that USP25 preferentially decreased K48‐linked ubiquitination of DDX58, whereas K63‐linked ubiquitination was unaffected (Figure 7G), indicating that USP25 specifically regulates K48‐linked ubiquitination of DDX58 via its enzymatic activity.

### 3.8 USP25 inhibits cardiomyocyte dysfunction via DDX58 stabilization

**FIGURE 8:**
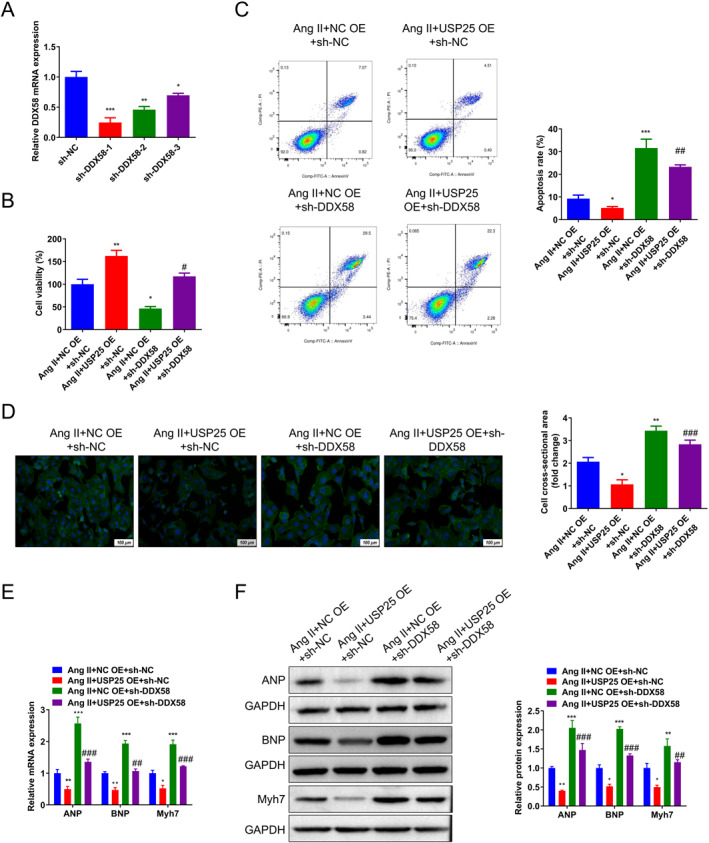
USP25 inhibits cardiomyocyte apoptosis and hypertrophy by stabilizing DDX58. To clarify whether USP25 exerts its regulatory effect on cardiomyocytes by stabilizing DDX58, AC16 cells were divided into four groups: Ang II + NC OE + sh‐NC group, Ang II + USP25 OE + sh‐NC group, Ang II + NC OE + sh‐DDX58 group, and Ang II + USP25 OE + sh‐DDX58 group. (A) Transfection efficiency of sh‐DDX58 shows that the sh‐DDX58‐1 has the highest transfection efficiency, so we chose the sh‐DDX58‐1 for the next experiments. (B) Cell viability detected by CCK‐8 assay. (C) Cell apoptosis detected by flow cytometry. (D) Cardiomyocyte volume detected by immunofluorescence staining. (E) Expression of pathological hypertrophy markers ANP, BNP and Myh7 detected by RT‐qPCR. (F) Expression of pathological hypertrophy markers ANP, BNP and Myh7 detected by western blot analysis. *, **, and *** represent *p* < 0.05, *p* < 0.01, and *p* < 0.001 compared to the Ang II + NC OE + sh‐NC group. ^#^, ^##^, and ^###^ represents *p* < 0.05, *p* < 0.01, and *p* < 0.001 compared to the Ang II + USP25 OE + sh‐NC group. ANP, A‐type natriuretic peptide; BNP, B‐type natriuretic peptide; NC, negative control; OE, overexpression; USP25, ubiquitin‐specific protease 25.

To determine whether the protective effects of USP25 depend on DDX58, AC16 cells were transfected with the USP25 OE vector alone or co‐transfected with sh‐DDX58. The sh‐DDX58‐1 construct, exhibiting the highest knockdown efficiency, was used for subsequent experiments (Figure 8A). USP25 OE alone enhanced cell viability (CCK‐8 assay, Figure 8B), reduced apoptosis (flow cytometry, Figure 8C), decreased cell hypertrophy (immunofluorescence staining, Figure 8D), and downregulated ANP, BNP, and Myh7 expression (RT‐qPCR and Western blot, Figure 8E,F). However, co‐silencing of DDX58 abrogated the protective effects of USP25, as all measured parameters (cell viability, apoptosis, and hypertrophy markers) returned toward baseline levels, indicating that the cardioprotective function of USP25 is dependent on DDX58.

### 3.9 miR‐339‐3p inhibition suppressed TAC‐induced ventricular remodeling in vivo

**FIGURE 9:**
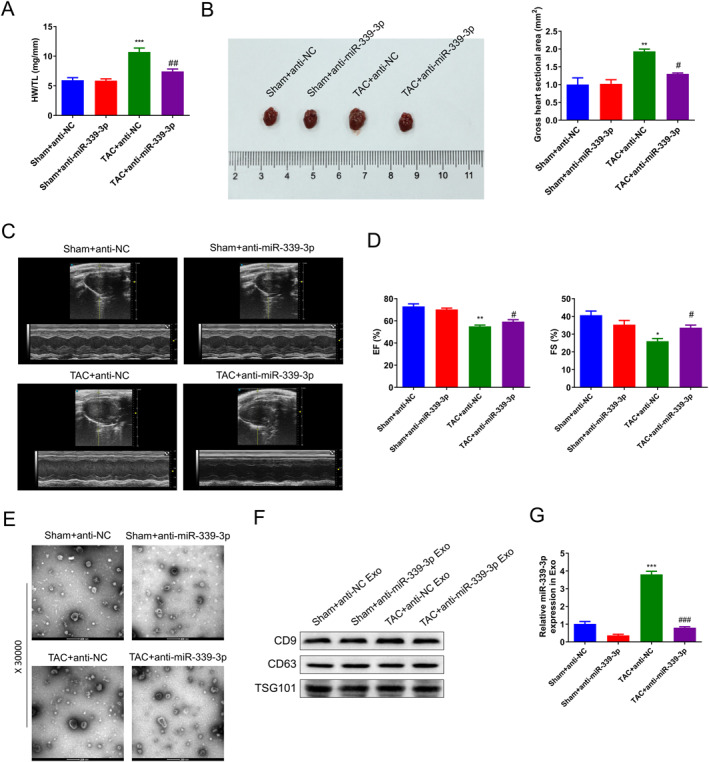
miR‐339‐3p inhibition alleviates TAC‐induced ventricular remodeling in in vivo experiments. To verify the regulatory effect of miR‐339‐3p on ventricular remodeling in vivo, a TAC‐induced mouse model of ventricular remodeling was established, and mice were divided into four groups: Sham + anti‐NC group, Sham + anti‐miR‐339‐3p group, TAC + anti‐NC group, and TAC + anti‐miR‐339‐3p group. (A) HW to TL ratio, showing that compared with the Sham + anti‐NC group, the TAC + anti‐NC group has a significantly increased HW/TL ratio. (B) Gross heart images of mice and quantification of gross heart sectional area, demonstrating that the TAC + anti‐NC group has an enlarged heart and increased sectional area compared to the Sham + anti‐NC group, while anti‐miR‐339‐3p reversed this change. (C) Echocardiographic images, revealing that the TAC + anti‐NC group exhibits abnormal cardiac structure and function compared to the Sham + anti‐NC group, while anti‐miR‐339‐3p makes these indicators return to normal. (D) EF and FS of mice detected by echocardiography, revealing that the TAC + anti‐NC group reduced EF and FS compared to the Sham + anti‐NC group, while anti‐miR‐339‐3p makes these indicators return to normal. (E) Circulating exosomes were collected from each group of mice (magnification: 30,000×). (F) Western blot analysis of exosomal marker proteins (CD9, CD63, and TSG101) in isolated exosomes. (G) Reverse transcription‐quantitative polymerase chain reaction analysis of relative miR‐339‐3p expression in circulating exosomes from mice in each group *, **, and *** represent *p* < 0.05, *p* < 0.01, and *p* < 0.001 compared to the negative control group. ^#^, ^##^, and ^###^represents *p* < 0.05, *p* < 0.01, and *p* < 0.001 compared to the model group. EF, ejection fraction; FS, fractional shortening; HW, heart weight; NC, negative control; TAC, transverse aortic constriction; TL, tibial length.

To validate the in vitro findings, a TAC mouse model was established, and anti‐miR‐339‐3p was administered to investigate the role of miR‐339‐3p in cardiac remodeling. TAC + anti‐NC animals exhibited an increased HW/TL ratio (Figure 9A), enlarged gross heart sectional area (Figure 9B), and abnormal echocardiographic parameters (Figure 9C,D), indicative of severe ventricular remodeling. Administration of anti‐miR‐339‐3p had no significant effect on cardiac parameters in sham‐operated mice, including HW/TL ratio, gross heart sectional area, and echocardiographic measurements (Figure 9A–D). In contrast, anti‐miR‐339‐3p markedly attenuated TAC‐induced ventricular remodeling, demonstrating that inhibition of miR‐339‐3p effectively mitigates TAC‐induced pathological cardiac changes.

Plasma exosomes were subsequently isolated from these mice. TEM revealed typical cup‐shaped exosome morphology, with no discernible differences in size or structure among the four experimental groups (Figure 9E). Western blot analysis confirmed the presence of canonical exosomal markers, including CD9, CD63, and TSG101 (Figure 9F). Furthermore, RT‐qPCR analysis indicated that miR‐339‐3p expression in exosomes was significantly elevated in the TAC + anti‐NC group compared with the Sham + anti‐NC group, and this upregulation was substantially reversed following anti‐miR‐339‐3p treatment (Figure 9G). These results confirm that miR‐339‐3p is present in circulating exosomes in TAC mice and that TAC‐induced ventricular remodeling is associated with the upregulation of exosomal miR‐339‐3p in vivo.

**FIGURE 10:**
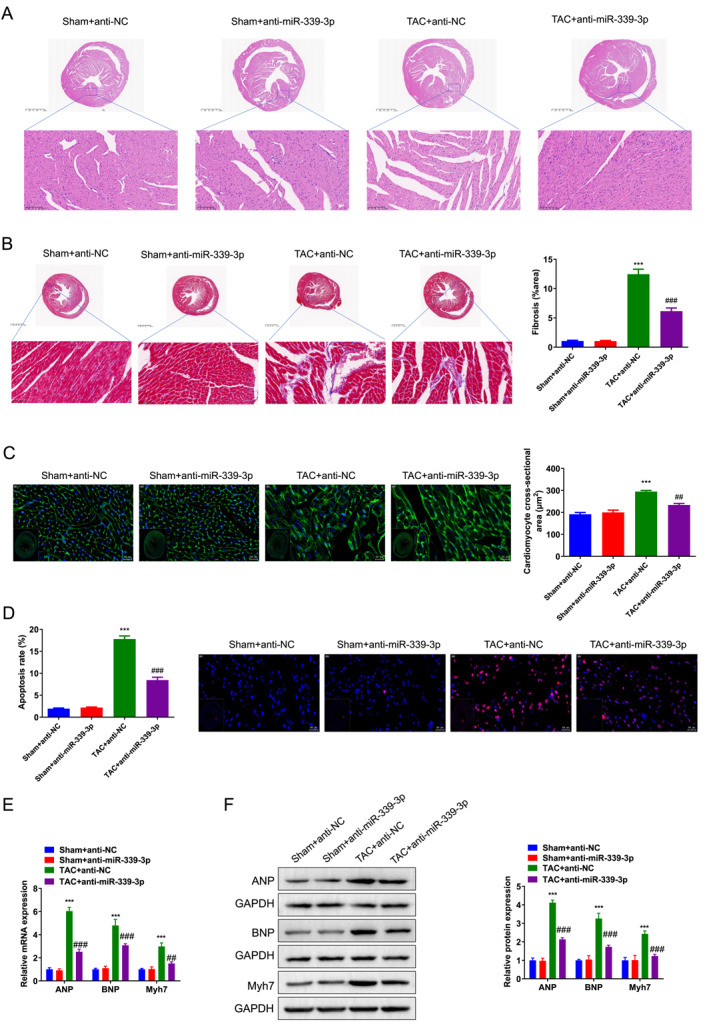
miR‐339‐3p inhibition suppressed TAC‐induced ventricular remodeling in in vivo experiments. To further confirm the effect of miR‐339‐3p on TAC‐induced ventricular remodeling, pathological staining and molecular detection were performed on the four groups of mice: Sham + anti‐NC, Sham + anti‐miR‐339‐3p, TAC + anti‐NC, TAC + anti‐miR‐339‐3p. (A) Hematoxylin‐eosin staining of heart tissue of different mice treated groups, the upper panels show low‐magnification images of heart sections, and lower panels show high‐magnification views of the corresponding areas to display detailed histological changes. (B) Myocardial fibrosis was assessed by Masson's trichrome staining. (C) Cardiomyocyte hypertrophy was evaluated by wheat germ agglutinin staining. (D) Cardiomyocyte apoptosis was detected by TUNEL staining. (E) mRNA expressions of ANP, BNP and Myh7 in different treated groups detected by RT‐qPCR. (F) Western blot results and quantification of protein expression of ANP, BNP and Myh7 in different treated groups detected by western blot analysis. *** represents *p* < 0.001 compared to the Sham + anti‐NC group. ^##^ and ^###^ represent *p* < 0.05 and *p* < 0.001 compared to the TAC + anti‐NC group. ANP, A‐type natriuretic peptide; BNP, B‐type natriuretic peptide; TAC, transverse aortic constriction; TUNEL, terminal deoxynucleotidyl transferase dUTP nick end labeling.

Histological analyses corroborated these findings. HE staining revealed characteristic pathological features of ventricular remodeling in the TAC + anti‐NC group, which were mitigated following anti‐miR‐339‐3p intervention (Figure 10A). Masson's trichrome staining demonstrated reduced myocardial collagen deposition (Figure 10B), WGA staining indicated decreased cardiomyocyte cross‐sectional area (*p* < 0.01; Figure 10C), and TUNEL staining showed a lower cardiomyocyte apoptosis rate (*p* < 0.001; Figure 10D) in the TAC + anti‐miR‐339‐3p group relative to the TAC + anti‐NC group. At both the mRNA (RT‐qPCR, Figure 10E) and protein (Western blot, Figure 10F) levels, the TAC + anti‐NC group exhibited elevated markers of ventricular remodeling compared with sham controls, whereas these changes were attenuated in the TAC + anti‐miR‐339‐3p group. These data indicate that inhibition of miR‐339‐3p suppresses TAC‐induced ventricular remodeling in vivo.

## 4 DISCUSSION

This study investigated the role of plasma exosomal miR‐339‐3p in myocardial remodeling associated with HF through both cellular and animal experiments. Our findings suggest that miR‐339‐3p may promote myocardial remodeling by targeting USP25, thereby modulating DDX58 deubiquitination. Plasma exosomes from HF patients exacerbated Ang II‐induced apoptosis and increased the cell surface area of AC16 cardiomyocytes, while simultaneously upregulating pathological hypertrophy markers ANP, BNP, and Myh7. These effects appeared to depend on the high expression of miR‐339‐3p in the exosomes, as confirmed by RNA sequencing. Subsequent bioinformatic predictions and experimental validation identified USP25 as a potential direct target of miR‐339‐3p. MiR‐339‐3p may negatively regulate USP25 expression, thereby attenuating its deubiquitination activity on the downstream interacting protein DDX58. These results were further corroborated using a TAC‐induced mouse model of ventricular remodeling. Inhibition of miR‐339‐3p might reduce the HW‐to‐tibial length ratio, alleviate left ventricular hypertrophy, improve cardiac function, and downregulate myocardial expression of ANP, BNP, and Myh7. Collectively, these findings indicate that exosomal miR‐339‐3p targets and suppresses USP25‐mediated DDX58 deubiquitination, thereby promoting cardiomyocyte apoptosis and hypertrophy and contributing to HF‐associated myocardial remodeling.

As critical “signal carriers” in cardiovascular pathophysiology, exosomes modulate target cell function through paracrine or autocrine mechanisms via their miRNA cargo. Previous studies have demonstrated that miR‐155‐5p in serum‐derived exosomes can exacerbate myocardial ischemia‐reperfusion injury by inhibiting NEDD4‐mediated CypD ubiquitination,^6^ while exosomes from patients with acute myocardial infarction promote endothelial angiogenesis via the miR‐126‐3p/TSC1/mTORC1/HIF‐1α pathway.^8^ Additionally, exosomes induced by remote ischemic preconditioning can deliver miR‐24 to attenuate myocardial ischemic injury.^18^ In the present study, plasma exosomes from HF patients were efficiently internalized by AC16 cardiomyocytes, significantly exacerbating Ang II‐induced apoptosis, hypertrophy, and upregulation of pathological markers (ANP, BNP, Myh7). Importantly, inhibition of miR‐339‐3p reversed these pathological effects, indicating that miR‐339‐3p might act as a key functional effector mediating myocardial injury within HF‐derived exosomes. These results are consistent with those of Galluzzo et al., ^11^ who reported that circulating miR‐339‐3p levels are associated with HF severity and NT‐proBNP concentrations. Further validation in hiPSC‐derived cardiomyocytes (hiPSC‐CMs) yielded highly consistent outcomes, reinforcing the robustness and reproducibility of our conclusion that exosomal miR‐339‐3p contributes to myocardial remodeling.

Notably, circulating exosomes in HF patients originate from diverse cellular sources, including cardiomyocytes and non‐cardiomyocyte cell populations. The elevated expression of exosomal miR‐339‐3p may be influenced by multiple factors, such as genetic background, environmental exposures (e.g., diet, medications, tissue injury, and surgical interventions), and the pathophysiological process of HF itself. Consequently, although exosomal miR‐339‐3p may not serve as the initial trigger of HF, it can exacerbate myocardial remodeling and accelerate disease progression.

A key mechanism in HF pathogenesis involves dysregulation of the ubiquitin–proteasome system, particularly via DUBs such as USP25. USP25, a member of the ubiquitin‐specific protease family, has been implicated in cardiovascular protection through substrate‐specific deubiquitination. Denuc et al. ^19^ demonstrated that USP25 undergoes both monoubiquitination and polyubiquitination at lysine 99 (K99), with these two modifications exerting opposing effects on its catalytic activity. Recent studies have identified USP25 as a critical regulator of myocardial remodeling: in Ang II‐ and TAC‐induced cardiac hypertrophy models, USP25 alleviates pathological remodeling by stabilizing SERCA2a, whereas in HF, it targets DDX58 (RIG‐I) to preserve its protein stability via K48‐linked deubiquitination. The multifunctionality of USP25 highlights its therapeutic potential, as it modulates two central HF pathways: calcium homeostasis and inflammatory signaling dysregulation. This is further supported by comprehensive reviews of DUBs in cardiovascular diseases, which confirm that USP25 stabilizes SERCA2a through K48‐linked deubiquitination, thereby improving calcium cycling and mitigating Ang II/TAC‐induced myocardial hypertrophy. Through bioinformatic prediction and dual‐luciferase reporter assays, USP25 was identified as a direct target of miR‐339‐3p. Subsequent experiments demonstrated that miR‐339‐3p significantly downregulates USP25 mRNA and protein expression, thereby impairing its deubiquitination function. Functional assays further showed that USP25 OE reverses myocardial injury induced by miR‐339‐3p mimics, indicating that USP25 is a key downstream effector of miR‐339‐3p in regulating myocardial remodeling. This finding expands the regulatory network of USP25 in HF, revealing its role as an effector molecule mediating the pathological effects of exosomal miRNAs.

DDX58, a critical receptor in innate immunity, has recently been implicated in ischemic HF. Yu et al.^20^ reported that DDX58 is highly expressed in ischemic HF and participates in immune dysregulation, with abnormalities in its protein stability exacerbating myocardial inflammation and injury. In the present study, STRING protein interaction prediction and Co‐IP experiments revealed a direct interaction between USP25 and DDX58. Furthermore, USP25 prolongs the half‐life of DDX58 and maintains its protein stability by reducing K48‐linked, but not K63‐linked, ubiquitination.^12^ Rescue experiments demonstrated that DDX58 knockdown completely abrogated the cardioprotective effects of USP25, indicating that DDX58 might be a critical substrate for USP25‐mediated cardiac protection. This observation aligns with prior findings showing that DUBs, such as BRCC3 and OTUD6a, regulate cardiovascular inflammation through substrate stabilization.^21^, ^22^ Collectively, our data extend this paradigm to the USP25–DDX58 axis in HF, highlighting the central role of immune dysregulation in myocardial remodeling and suggesting that the USP25–DDX58 pathway represents a key deubiquitinase‐mediated mechanism in HF‐related inflammation.^23^, ^24^

DDX58 (RIG‐I) is a classical cytosolic RNA sensor that mediates innate immune and inflammatory responses through activation of NF‐κB and type I interferon signaling pathways, both of which are widely recognized as critical drivers of myocardial inflammation and pathological remodeling. Recent studies have confirmed that DDX58 is significantly upregulated in failing hearts and is closely associated with cardiomyocyte apoptosis, ventricular dysfunction, and adverse cardiac remodeling. Notably, emerging evidence indicates that the functional role of DDX58 is highly context‐dependent and cell type‐specific. In cardiomyocytes, the stability of DDX58 protein can directly regulate apoptosis and hypertrophy independently of classical inflammatory signaling pathways. In this study, USP25 enhanced DDX58 stability by cleaving K48‐linked ubiquitination, and DDX58 protected cardiomyocytes against Ang II‐induced injury.^12^, ^20^ These findings extend the regulatory network of the USP25–DDX58 axis in myocardial remodeling and suggest that DDX58 may exert a cardioprotective role that is distinct from its pro‐inflammatory function in immune cells.

In the mouse model of TAC‐induced ventricular remodeling, inhibition of miR‐339‐3p significantly reduced the HW/TL ratio, improved left ventricular EF, downregulated the expression of myocardial hypertrophy markers, and restored USP25 and DDX58 protein levels. These results are fully consistent with the in vitro experimental findings, confirming the pathological relevance of the plasma exosomal miR‐339‐3p–regulated USP25–DDX58 pathway in vivo. Previous study demonstrated that inhibition of DUBs such as OTUD1 or activation of USP25 alleviates TAC‐induced hypertrophy and cardiac dysfunction, which aligns with our observation that miR‐339‐3p inhibition exerts protective effects.^23^

Despite elucidating the regulatory role of the plasma exosomal miR‐339‐3p–USP25–DDX58 axis in myocardial remodeling in HF, several areas warrant further investigation. Firstly, this study included plasma samples from only five HF patients and five healthy controls for exosome extraction and miRNA screening, representing a relatively small sample size. Moreover, the study did not differentiate between HF etiologies. Future studies should expand the sample size and perform subgroup analyses to validate the expression patterns and clinical significance of this axis in patients with diverse HF phenotypes. Secondly, this study focused exclusively on the K48‐linked deubiquitination of DDX58 by USP25; whether additional post‐translational modifications contribute to this axis remains unclear. Additionally, beyond miR‐339‐3p, other differentially expressed miRNAs or proteins within exosomes may collaborate to regulate myocardial remodeling in HF. Further research should explore additional regulatory molecules to construct a more comprehensive network of exosome‐mediated pathogenic mechanisms and include in vivo experiments to directly validate these pathways, thereby enhancing research rigor.

## 5 CONCLUSION

In conclusion, this study demonstrates that plasma exosomal miR‐339‐3p targets USP25 and inhibits its K48‐linked deubiquitination of DDX58, resulting in increased ubiquitination and degradation of DDX58. This process exacerbates cardiomyocyte apoptosis and hypertrophy, ultimately promoting HF‐associated myocardial remodeling. Collectively, these findings indicate that plasma exosomal miR‐339‐3p may serve as a potential biomarker for evaluating HF severity and prognosis, whereas USP25 and DDX58 represent promising therapeutic targets, providing an experimental basis for the development of precise treatment strategies for HF.

## AUTHOR CONTRIBUTIONS

Yuhong Ma designed and supervised the study. Guoqiang Jing and Ting Xu wrote the original draft and conducted experiments. Guoqiang Jing and Ting Xu collected raw data. Guoqiang Jing performed statistical and bioinformatics analyses.

## CONFLICT OF INTEREST STATEMENT

The authors declare no conflicts of interest.

## ETHICS STATEMENT

This study was approved by the Ethics Committee of Affiliated Hospital of Inner Mongolia Medical University and all participants provided informed consent.

## Supporting information

Supporting Information S1

Figure S1

Figure S2

## Data Availability

The data that support the findings of this study are available from the corresponding author upon reasonable request.
