# Supplementary material for: Plasma exosomal miR‐339‐3p promotes myocardial remodeling in chronic heart failure by regulating USP25‐mediated DDX58 deubiquitination
Source: J Cell Commun Signal. 2026 Jun 26;20(3):e70090. doi: 10.1002/ccs3.70090 (PMC13309614; doi:10.1002/ccs3.70090)
Supplement: Supplementary file 1 — Supporting Information S1 [file CCS3-20-e70090-s001.docx]

**Figure S1 GO and KEGG pathway analysis of differently expressed HF-exo miRNAs between healthy controls and HF patients**

1. Gene ontology analysis (GO analysis); B. Kyoto encyclopedia of genes and genomes analysis (KEGG analysis). All enrichments results were filtered with *P* value < 0.05, the y-axis represents the names of GO terms or KEGG pathways, and the x-axis represents the -log_10_ (*P* value), where a larger value indicates a more significant enrichment.

**Figure S2**. Silencing miR-339-3p alleviates the promoting effects of exosomes from heart failure patients on apoptosis and hypertrophy in hiPSC-derived cardiomyocytes.

hiPSC-CMs were treated with Ang II, Ang II + HF-exo + NC inhibitor, or Ang II + HF-exo + miR-339-3p inhibitor; A. Transfection efficiency of miR-339-3p inhibitor was verified by RT-qPCR. B. The expression level of miR-339-3p in hiPSC-CMs was detected by RT-qPCR; C. Cell viability was assessed by CCK-8 assay; D. Cell apoptosis was detected by flow cytometry; E. Cardiomyocyte hypertrophy was evaluated by FITC-phalloidin staining (scale bar, 20 µm); F. The mRNA expression levels of hypertrophic markers (ANP, BNP, Myh7) were detected by RT-qPCR. G. The protein expression levels of hypertrophic markers (ANP, BNP, Myh7) were detected by Western blot. ^*^*P* < 0.05, ^**^*P* < 0.01, ^***^*P* < 0.001 vs. Ang II group; ^#^*P* < 0.05, ^##^*P* < 0.01 vs. Ang II + HF-exo + NC inhibitor group.
