# Supplementary figures and images for: Plasma exosomal miR‐339‐3p promotes myocardial remodeling in chronic heart failure by regulating USP25‐mediated DDX58 deubiquitination
Source: J Cell Commun Signal. 2026 Jun 26;20(3):e70090. doi: 10.1002/ccs3.70090 (PMC13309614; doi:10.1002/ccs3.70090)

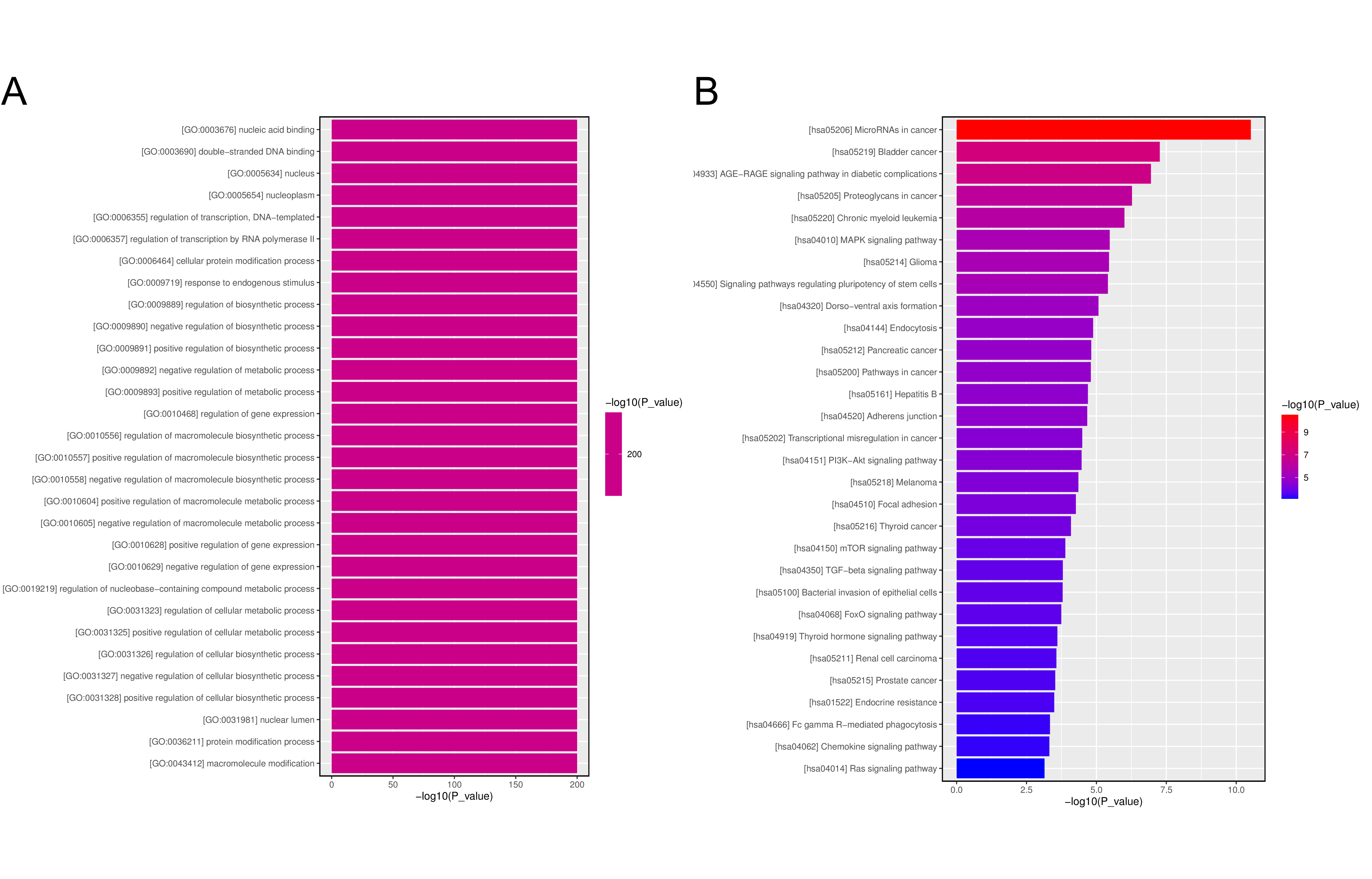

Supplement: Supplementary file 2 — Figure S1 [file CCS3-20-e70090-s003.jpg]

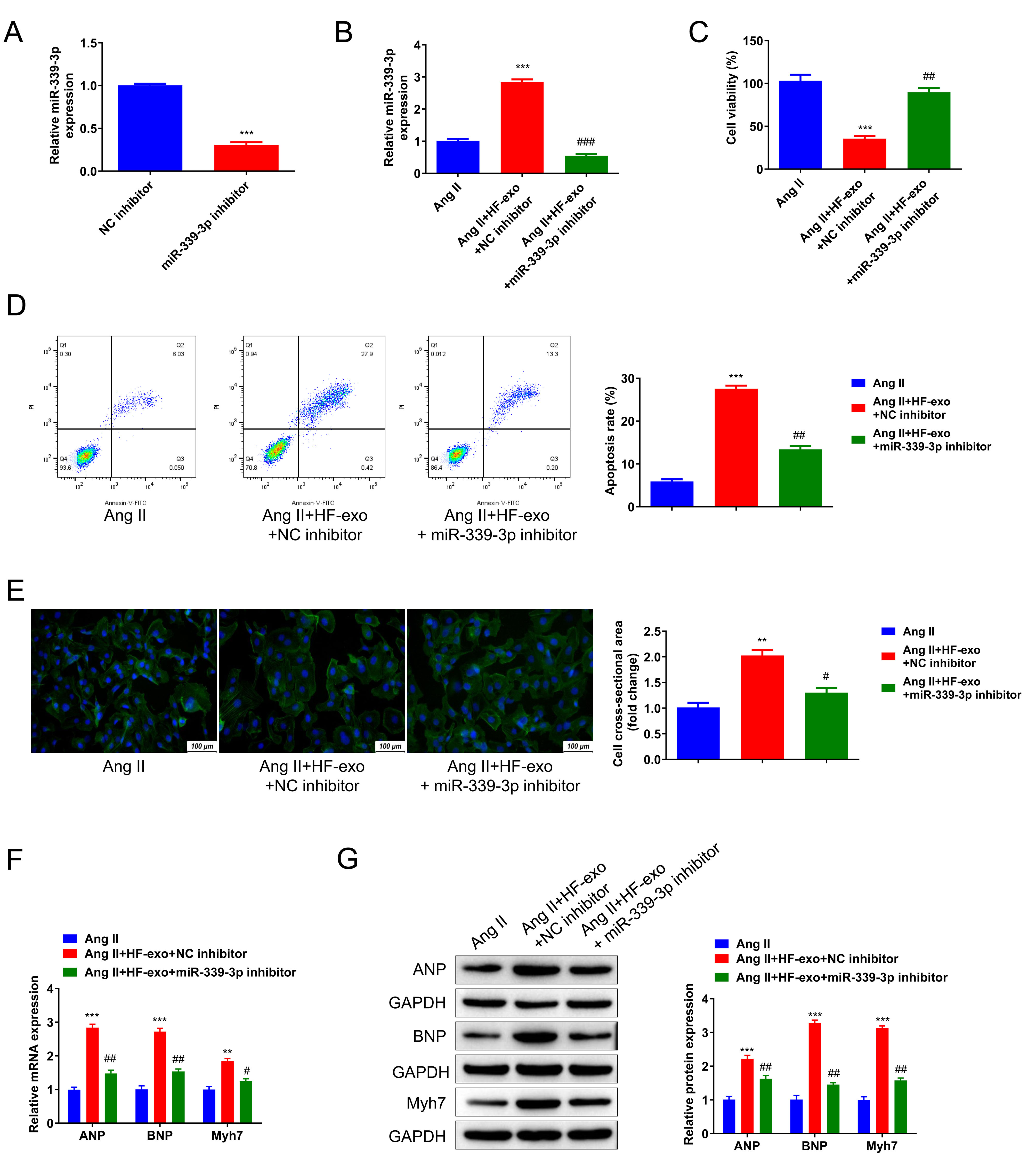

Supplement: Supplementary file 3 — Figure S2 [file CCS3-20-e70090-s002.jpg]
